# Improvement of depression in a patient with hypothyroidism and deiodinase polymorphism with LT3 Therapy

**DOI:** 10.1002/ccr3.5651

**Published:** 2022-04-12

**Authors:** Ziyan S. Ahmed, Rinsha P. V. Sherin, Tatiana L. Fonseca, Thanh D. Hoang, Mohamed K. M. Shakir

**Affiliations:** ^1^ 8395 Endocrinology Division Walter Reed National Military Medical Center Bethesda Maryland USA; ^2^ Section of Adult and Pediatric Endocrinology Diabetes & Metabolism University of Chicago Chicago Illinois USA; ^3^ Endocrinology Division Uniformed Services University of the Health Sciences Bethesda Maryland USA

**Keywords:** depression, hypothyroidism, polymorphism, T3

## Abstract

We report a 54‐year‐old man with treatment‐resistant depression (TRD) and hypothyroidism who responded to LT3/LT4 combination, rather than LT4 alone. He was able to discontinue all antidepressant medications eventually. Interestingly, the patient has a DIO2 polymorphism.

## Graphical abstract

Patients with treatment‐resistant depression (TRD) often need an augmentation therapy. In this report, our patient with TRD, hypothyroidism, and Thr92 Ala‐DIO2 polymorphism had responded well to T3 therapy.

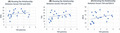

## 1 INTRODUCTION

A relationship between hypothyroidism and depression has been assumed for many years; however, the true nature of this association has been difficult to demonstrate. However, our knowledge in this area has expanded significantly with large cohort studies and genetically driven studies being published.

Treatment‐resistant depression (TRD) traditionally refers to inadequate clinical response following the antidepressant therapy ^1^, ^2^, ^3^. Patients with depression who fail to achieve symptom remission may pose significant clinical challenges especially when associated with hypothyroidism and need an augmentation pharmacology approach ^4^, ^5^. We report a patient with TRD, hypothyroidism, and Thr92 Ala‐DIO_2_ polymorphism who has responded well to T3 therapy in the form of augmentation therapy, given in 3 divided doses.

## 2 METHODS

The general health questionnaire (GHQ), thyroid symptom questionnaire (TSQ) and Beck Depression GHQ Inventory (BDI) assessments.

The GHQ is a well‐validated screening tool designed to evaluate mood and anxiety disorders. The higher the score from the GHQ, the more symptomatic the patient is.

The TSQ assesses any persisting symptoms in hypothyroid patients who are on thyroid hormone treatment to detect any significant psychological impairment and hypothyroid symptoms in comparison with euthyroid subjects. The higher the score from the TSQ, the more symptomatic the patients are.

The BDI is a 21‐item self‐report rating assessment that measures characteristic attitudes and symptoms of depression. Total BDI scores of 1‐10 are considered normal; 11‐16 suggest mild mood disturbance; 17‐20 borderline clinical depression; 21‐30 moderate depression; 31‐40 severe depression; and over 40 extreme depression. ^6^, ^7^, ^8^

Due to his ongoing TRD, he was followed only with GHQ, TSQ, BDI‐II; and the Wechsler Memory Scale test could not be performed.

### 2.1 Laboratory assessments

Routine laboratory tests including thyroid functions were performed at the Walter Reed National Military Medical Center, Bethesda, Maryland. Genotyping was performed as per the Allelic Discrimination protocol from a real‐time PCR machine (Applied Biosciences) using TaqMan^TM^ reagents and rs225014 SNP primer. ^9^, ^10^

### 2.2 Statistical analysis

A statistical analysis was performed through an analysis of variance (ANOVA) test, and a post hoc analysis was performed through a Tukey test.

## 3 CASE REPORT

**FIGURE 1:**
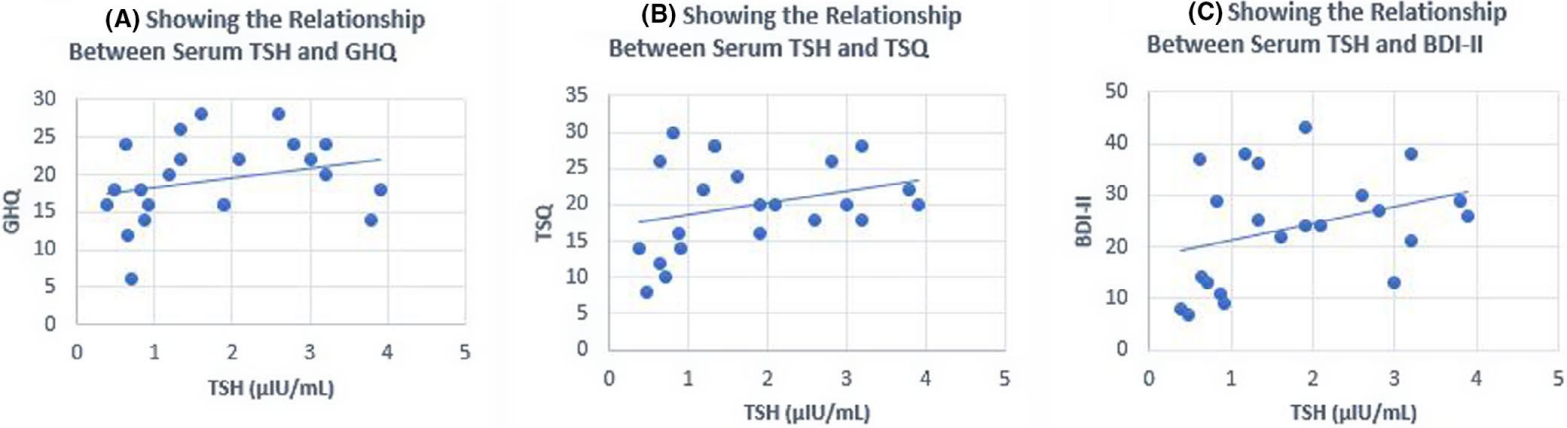
Relationship between serum TSH (thyroid stimulating hormone) and GHQ, TSQ, and BDI‐II

**FIGURE 2:**
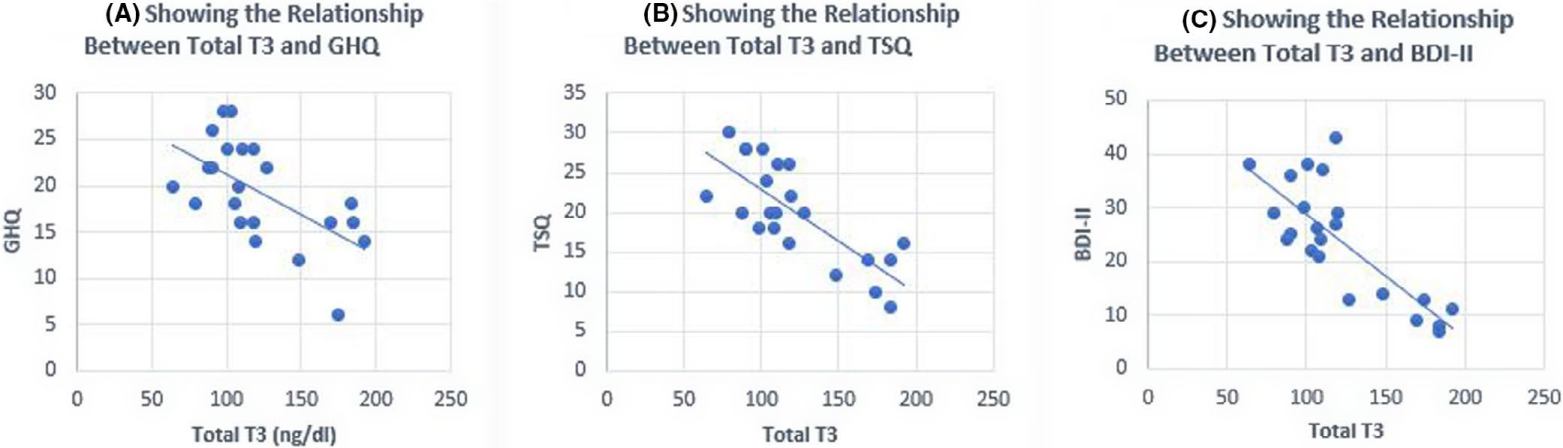
Relationship between serum total T3 (triiodothyronine) levels and GHQ, TSQ, and BDI‐II

**FIGURE 3:**
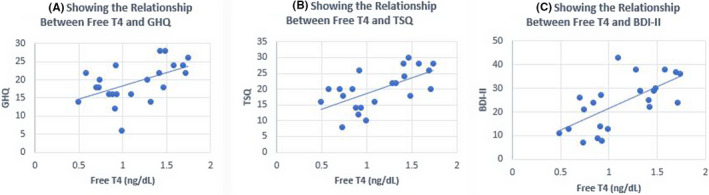
Relationship between free T4 (thyroxine) levels and GHQ, TSQ, and BDI‐II. Beck Depression Inventory‐II (BDI‐II), consisting of 21 items assessing specific cognitive, affective, and physical symptoms of depression, corresponds well to a clinical diagnosis of depressive disorders outlined in the Diagnostic and Statistical Manual of Mental Disorders, Fourth Edition (8–10). Total BDI scores of 1–10 are considered normal. Scores of 11–16 suggest mild mood disturbance; 17–20 (borderline clinical depression); 21–30 (moderate depression); 31–40 (severe depression); over 40 (extreme depression). Validity of these tests in patients with hypothyroidism has been well established previously (8.9). Serum TSH, free T4, and total T3 were measured by the electrochemiluminescence immunoassay (ECLIA, Cobas 8000, K Diagnostics, Indianapolis, IN). DNA was extracted from blood samples

A 54‐year‐old man presented to the emergency room with suicidal ideation 5 years ago. In addition, the patient had severe depression and an immense desire to surf generally prohibited internet sites. He also noted a 12‐pound weight gain, dry skin, and cold intolerance. Family history was significant for Hashimoto’s thyroiditis in one daughter. His physical examination revealed: heart rate 64 bpm, blood pressure 140/90 mm Hg. His thyroid was diffusely enlarged, approximately 40 grams without palpable nodules. However, the rest of the examination was normal with the exception of delayed deep tendon reflexes. Laboratory values at admission showed normal CBC, CMP, and serum B12 level. Thyroid functions showed serum TSH 560 µIU/mL (normal 0.41–4.2), FT4 0.20 ng/dL (normal 0.90–2.18), total T3 38 ng/dL (normal 59–174), TPO antibody 278 IU/mL (normal 0–34), and TG antibody 9.8 ng/dL (normal 0.0–0.9). An ultrasound confirmed a heterogeneously enlarged thyroid consistent with Hashimoto’s thyroiditis. A diagnosis of major depressive disorder and primary hypothyroidism were made. The GHQ, TSQ, and BDI‐II scores obtained 2 days after admission were significantly abnormal (Table 1). During the 8 week basal period, the symptom scores and thyroid functions were measured at 3 week intervals (*n* = 4) and these functions were GHQ 26.0 ± 8.49, TSQ 28.5 ± 7.77, BDI‐II 45.0 ± 6.18, TSH 165 ± 133 µIU/mL, free T4 0.79 ± 0.25 ng/dL, and total T3 60.5 ± 7.89 ng/Dl ((Table 1). The patient was treated with daily doses of 175 mcg of levothyroxine (LT4) and 20 mg of citalopram. The daily dose of citalopram was gradually increased to 40mg and the patient additionally was prescribed amitriptyline 50 mg at bedtime along with bi‐weekly psychotherapy sessions. After 8 weeks of treatment, there was modest improvement in GHQ, TSQ, and BDI‐II despite normal serum TSH 1.34 µIU/mL (Table 1, Figures 1,2). Three months later, because of the poor response to antidepressant drugs, aripiprazole and buspirone were added. Since patient required intense psychotherapy and a regimen of 4 different antidepressant medications, a diagnosis of TRD was confirmed. After 9 months, a daily dose of 5 mcg of LT3 was added. The patient continued LT4 + LT3 combination for another 13 months, but this time, the treatment was changed to LT4 only. Despite being on 4 antidepressant drugs and LT4, his depression scores and internet addiction showed no improvement (Table 1). Approximately 2 ½ years after the initial visit, patient was placed on a thrice daily dosage of 5 mcg of LT3 with reduced dose of LT4. Within 4 weeks of starting LT4 + LT3 TID dosing, patient noted remarkable improvement in depression, suicidal ideation, and internet addiction. It was possible to gradually discontinue all the antidepressant drugs over the next 9 months (Table 1); however, the psychiatrist prescribed over‐the‐counter antidepressant drugs, S‐adenosylmethionine (SAMe), (Pure Encapsulations ^TM^, Sudbury MA) 1600 mg/day, and Rhodiola rosea (rosavins >3.0%, salidroside >0.8% at a ratio of 3:1, Pure Encapsulations ^TM^, Sudbury MA) 200 mg twice daily. The serum total T3 levels remained in the upper normal range while the patient was taking LT4 + LT3 5 mcg TID dosing and the serum TSH levels remained in the lower normal range. A follow‐up cardiac evaluation was completely normal. He presently continues psychotherapy visits only once every 3 months. In addition, he is gainfully employed and also continues a happy married life. A genetic screen performed for DIO2 polymorphism confirmed that the patient carries one allele of Thr92 Ala‐DIO_2_ polymorphism. The GHQ, TSQ, and BDI‐II scores correlated well with serum TSH and T3 levels (Figures 1,2), but less significant with serum free T4 levels (correlation coefficient (r) for GHQ: 0.52, r for TSQ: 0.62, r for BDI‐II 0.66, Figures 3).

**TABLE 1:** Following admission to psychiatry ward patient received psychotherapy, antidepressant drugs, and levothyroxine

Periods	Treatment	Months (Duration)	n	GHQ	TSQ	BDI‐II	TSH (µIU/mL)	Free T4 (ng/dL)	Total T3 (ng/dL)
1	T4 Only	9	6	23.0 ± 1.53	26.3 ± 1.20	31.2 ± 2.77	1.15 ± 0.36	1.50 ± 0.07	89.33 ± 6.74
2	T4 and T3 QD	12	5	20.0 ± 1.41	20.8 ± 1.35	22.2 ± 2.52	2.96 ± 0.32	0.76 ± 0.06	113.6 ± 3.93
3	T4 Only	8	5	20.8 ± 2.58	20.8 ± 2.05	32.8 ± 3.40	2.72 ± 0.35	1.44 ± 0.11	104.8 ± 5.99
4	T4 and T3 TID	30	6	13.7 ± 1.74	12.3 ± 1.20	10.3 ± 1.14	0.67 ± 0.09	0.82 ± 0.08	175.0 ± 6.32

Note

During the 8 week basal period, the symptom scores and thyroid functions were measured at 2–3 weeks intervals (*n* = 4) and these functions were GHQ, 26.0 ± 8.49, TSQ 28.5 ± 7.77, BDI‐II 45.0 ± 6.18, TSH 165 ± 133 µIU/mL, Free T4 0.79 ± 0.25 ng/dL, and total T3 60.5 ± 7.89 ng/dL ( not shown in the Table). After achieving euthyroid status (serum TSH 1.34 µIU/mL, Free T4 1.74 ng/dL), the symptom scores and thyroid functions were measured at 4–8 weeks while patient was receiving levothyroxine only (period 1), levothyroxine + liothyronine QD (once daily) (period 2), levothyroxine only (period 3, 2nd time), and levothyroxine and liothyronine TID (three times daily, period 4). All values are shown as mean ± SEM.

Abbreviations: Periods 1,2,3,4 when various thyroid medication were used, duration = number of months, the patient was on medication n=number of measurements, GHQ = general health questionnaire, TSQ = thyroid symptom questionnaire. BDI‐II Beck Depression Inventory‐II, TSH = thyroid stimulating hormone, Free T4 = free thyroxine, T3 = triiodothyronine. The validity of GHQ, TSQ, and BDI‐II measurements has been previously established ^7^, ^8^. Serum TSH, Free T4, and total T3 were measured by the electrochemiluminescence immunoassay (ECLIA, Cobas 8000, K Diagnostics, Indianapolis, IN).

## 4 DISCUSSION

It has been clearly demonstrated that some subjects with hypothyroidism even when treated have poorer well‐being compared with the general population. It also appears that the association between thyroid function and depression is more clearly seen in this population. Furthermore, the management of patients presenting with hypothyroidism and TRD is challenging. While there is no universally agreed upon definition of TRD ^4^, it is well‐accepted that patients with TRD have a poor prognosis. Although our patient was treated with four antidepressant drugs, there was an inadequate response to his depression and internet addiction. With a diagnosis of primary hypothyroidism, the patient was treated with a weight‐based dose of levothyroxine as recommended by the American Thyroid Association ^11^ and the patient achieved euthyroid status within 6 weeks and his serum T3 levels were in the normal range. It has been previously noted that a significant proportion of patients with hypothyroidism treated with levothyroxine, the serum T3 levels may remain in the low normal range and the exact significance of this is not known ^12^. Finally, when our patient received LT3 in 3 divided doses, in the form of augmentation therapy there was a significant improvement in his depressive symptoms and internet addiction, along with improvement in his GHQ, TSQ and BDI‐II scores. However, it was noted that his serum TSH levels were in low normal range. Our patient had no signs/symptoms of hyperthyroidism and had a normal cardiac screening. Finally, he was able to discontinue all prescribed antidepressant drugs, and was treated with 2 over‐the‐counter drugs namely S‐adenosylmethionine (SAMe) ^13^ and Rhodiola Rosea supplements ^14^. In addition, our patient required psychotherapy sessions less frequently. Thus, the LT4 + LT3 TID, SAMe, and Rhodiola Rosea combination therapy resulted in significant improvement in his major depressive disorder and the associated internet addiction, an obsessive neurosis and he is able to lead a normal life. There have been considerable controversies regarding what constitutes TRD. However, the inadequate response to trials of 4 antidepressant drugs met to criteria of TRD in our patient. In contrast to the well‐accepted treatment of hypothyroidism with LT4, LT3 is often prescribed in depression as an augmentation pharmacotherapy ^15^. Limited studies utilizing LT3 therapy were conducted as an augmentation of tricyclic antidepressant and found improvement in depression ^15^, ^16^. Open label studies have also shown some benefits ^16^. However, there was no superior benefit compared with lithium, although the side effects were much less ^16^. Once daily administration of short‐acting commercially available T3 preparation in hypothyroid individuals would not be predicted to be associated with steady T3 levels based on pharmacokinetic data. Although there is interest in developing a sustained release T3 preparation that maintains stable serum concentrations of T3, currently this is not available and hence, it is suggested that T3 at a lower dose of 5 mcg thrice daily may be a preferred form of augmentation therapy. Further studies involving large number of patients are needed

Thyroid hormone may play a significant role in noradrenergic and serotonergic neurotransmission as well as in the pathogenesis of depression. However, screening patients with depression for hypothyroidism is not widely supported ^17^. Previous studies ^18^, ^19^ have shown an association between immune thyroid diseases and depression, although in one study of subjects with normal thyroid function, there was no association between TPO antibodies and depression ^20^. Our patient had markedly elevated thyroid antibodies, and it is not clear whether there was any association between the antibodies and depression.

The use of LT4/LT3 combination therapy or LT3 treatment alone in depression remains controversial. Panicker et al ^21^ showed that patients with a functional polymorphism in DIO_2_ may respond better with a LT4/LT3 combination treatment. Compared with previous studies in which T3 supplementation has not been beneficial, our patient differs in several ways. Our patient had complete failure of thyroid gland as evidenced by low T4 levels, and the need for full body weight adjusted replacement dose of LT4. It is also unlikely that the improvement resulted from a placebo effect for several reasons. Our patient had already taken T4 alone on 2 occasions and LT4 + LT3 once daily previously without improvement. In addition to his subjective sense of wellness, the GHQ, TSQ, and BDI II scores used as measures of improvements in hypothyroid patients from our institution ^6^, ^7^ also confirmed benefits on several occasions. Finally, our patient had well‐documented TRD. However, re‐challenging the patient with a higher dose of LT4 alone to keep the TSH in the low normal range would have confirmed this, but we felt it was unethical. In addition, since our patient noted significant improvements, he would have refused any modification of the current treatment utilizing LT4 + LT3 combination. Since there is only D_2_ in the brain which converts LT4 to LT3, it is possible that when hypothyroid patients with Thr92 Ala‐DIO_2_ polymorphism is treated with a thrice daily LT3 regimen, they may respond much better as evidenced by improvements in TRD and various scores. In patients with carriers of the Thr92Ala‐DIO_2_ polymorphism, there may be subtle changes in thyroid hormone homeostasis and accumulation of Ala92‐D_2_ in the trans‐Golgi apparatus providing less T3 locally ^22^. These patients may be at higher risk for brain degenerative disease even if they maintain euthyroid. It is possible that the steady state of serum T3 levels in the high normal range may have been able to at least partially overcome the monocarboxylate transporter (MCT) system especially MCT8 as suggested by Jo et al ^23^.

Patients with depression who fail to achieve symptom remission in a timely fashion may pose significant clinical challenges and often need an augmentation therapy. In this case report, a patient with TRD, hypothyroidism, and Thr92 Ala‐DIO2 polymorphism had responded well to T3 therapy given in three divided doses. There was a good correlation between the depression scores and the serum T3 levels. In hypothyroid patients with functional DIO2 polymorphism, thrice daily LT3 dosing given as a form of augmentation therapy may significantly improve depression. Additional studies involving large number of patients are needed.

## CONFLICT OF INTEREST

None to declare.

## AUTHOR CONTRIBUTION

ZSA is the author of the manuscript. RPVS, TDH, and MKMS are the reviewers of the manuscript. TLF is the reviewer and genetic researcher of the manuscript.

## ETHICAL APPROVAL

The manuscript has been reviewed and approved by the IRB and Public Affairs Office.

## CONSENT

The authors have confirmed that patient consent has been signed and collected in accordance with the journal’s patient consent policy.

## Data Availability

Not applicable.

## References

[1] Ittermann T , Völzke H , Baumeister SE , Appel K , Grabe HJ . Diagnosed thyroid disorders are associated with depression and anxiety. Soc Psychiatry Psychiatr Epidemiol. 2015;50(9):1417‐1425. 25777685 10.1007/s00127-015-1043-0

[2] Demartini B , Masu A , Scarone S , Pontiroli AE , Gambini O . Prevalence of depression in patients affected by subclinical hypothyroidism. Panminerva Med. 2010;52(4):277‐282. 21183887

[3] Chueire VB , Romaldini JH , Ward LS . Subclinical hypothyroidism increases the risk for depression in the elderly. Arch Gerontol Geriatr. 2007;44(1):21‐28. 16678286 10.1016/j.archger.2006.02.001

[4] Ruberto VL , Jha MK , Murrough JW . Pharmacological treatments for patients with treatment‐resistant depression. Pharmaceuticals (Basel). 2020;13(6):E116. 32512768 10.3390/ph13060116 PMC7345023

[5] Lam RW , Hossie H , Solomons K , Yatham LN . Citalopram and bupropion‐SR: combining versus switching in patients with treatment‐resistant depression. J Clin Psychiatry. 2004;65(3):337‐340. 15096072

[6] Clyde PW , Harari AE , Getka EJ , Shakir KMM . Combined levothyroxine plus liothyronine compared with levothyroxine alone in primary hypothyroidism: a randomized controlled trial. JAMA. 2003;290(22):2952‐2958. 14665656 10.1001/jama.290.22.2952

[7] Hoang TD , Olsen CH , Mai VQ , Clyde PW , Shakir MK . Desiccated thyroid extract compared with levothyroxine in the treatment of hypothyroidism: a randomized, double‐blind, crossover study. J Clin Endocrinol Metab. 2013;98(5):1982‐1990. 23539727 10.1210/jc.2012-4107

[8] Beck AT , Steer RA , Ball R , Ranieri W . Comparison of beck depression inventories ‐IA and ‐II in psychiatric outpatients. J Pers Assess. 1996;67(3):588‐597. 8991972 10.1207/s15327752jpa6703_13

[9] Peeters RP , van Toor H , Klootwijk W , et al. Polymorphisms in thyroid hormone pathway genes are associated with plasma TSH and iodothyronine levels in healthy subjects. J Clin Endocrinol Metab. 2003;88(6):2880‐2888. 12788902 10.1210/jc.2002-021592

[10] Mentuccia D , Proietti‐Pannunzi L , Tanner K , et al. Association between a novel variant of the human type 2 deiodinase gene Thr92Ala and insulin resistance: evidence of interaction with the Trp64Arg variant of the beta‐3‐adrenergic receptor. Diabetes. 2002;51(3):880‐883. 11872697 10.2337/diabetes.51.3.880

[11] Jonklaas J , Bianco AC , Bauer AJ , et al. Guidelines for the treatment of hypothyroidism: prepared by the american thyroid association task force on thyroid hormone replacement. Thyroid. 2014;24(12):1670‐1751. 25266247 10.1089/thy.2014.0028 PMC4267409

[12] Gullo D , Latina A , Frasca F , Le Moli R , Pellegriti G , Vigneri R . Levothyroxine monotherapy cannot guarantee euthyroidism in all athyreotic patients. PLoS One. 2011;6(8):e22552. 21829633 10.1371/journal.pone.0022552 PMC3148220

[13] Cuomo A , Beccarini Crescenzi B , Bolognesi S , et al. S‐Adenosylmethionine (SAMe) in major depressive disorder (MDD): a clinician‐oriented systematic review. Ann Gen Psychiatry. 2020;19:50. 32939220 10.1186/s12991-020-00298-z PMC7487540

[14] Konstantinos F , Heun R . The effects of rhodiola rosea supplementation on depression, anxiety and mood – a systematic review. Global Psychiatry. 2020;3:72‐82. doi:10.2478/gp-2019-0022

[15] Kelly TF , Lieberman DZ . Long term augmentation with T3 in refractory major depression. J Affect Disord. 2009;115(1‐2):230‐233. 19108898 10.1016/j.jad.2008.09.022

[16] Nierenberg AA , Fava M , Trivedi MH , et al. A comparison of lithium and T(3) augmentation following two failed medication treatments for depression: a STAR*D report. Am J Psychiatry. 2006;163(9):1519‐1530. 16946176 10.1176/ajp.2006.163.9.1519

[17] Kim JS , Zhang Y , Chang Y , et al. Subclinical hypothyroidism and incident depression in young and middle‐age adults. J Clin Endocrinol Metab. 2018;103(5):1827‐1833. 29408972 10.1210/jc.2017-01247

[18] Pop VJ , Maartens LH , Leusink G , et al. Are autoimmune thyroid dysfunction and depression related. J Clin Endocrinol Metab. 1998;83(9):3194‐3197. Kirim S, Keskek SO, Köksal F, 9745425 10.1210/jcem.83.9.5131

[19] Haydardedeoglu FE , Bozkirli E , Toledano Y . Depression in patients with euthyroid chronic autoimmune thyroiditis. Endocr J. 2012;59(8):705‐708. 22673294 10.1507/endocrj.ej12-0035

[20] Engum A , Bjøro T , Mykletun A , Dahl AA . Thyroid autoimmunity, depression and anxiety; are there any connections? an epidemiological study of a large population. J Psychosom Res. 2005;59(5):263‐268. 16253615 10.1016/j.jpsychores.2005.04.002

[21] Panicker V , Saravanan P , Vaidya B , et al. Common variation in the DIO2 gene predicts baseline psychological well‐being and response to combination thyroxine plus triiodothyronine therapy in hypothyroid patients. J Clin Endocrinol Metab. 2009;94(5):1623‐1629. 19190113 10.1210/jc.2008-1301

[22] McAninch EA , Rajan KB , Evans DA , et al. A common DIO2 polymorphism and alzheimer disease dementia in African and European Americans. J Clin Endocrinol Metab. 2018;103(5):1818‐1826. 29481662 10.1210/jc.2017-01196 PMC6276710

[23] Jo S , Fonseca TL , Bocco BMLC , et al. Type 2 deiodinase polymorphism causes ER stress and hypothyroidism in the brain. J Clin Invest. 2019;129(1):230‐245. 30352046 10.1172/JCI123176 PMC6307951

